# Hepatic Arterial Infusion Chemotherapy for Unresectable Intrahepatic Cholangiocarcinoma, a Comprehensive Review

**DOI:** 10.3390/jcm10122552

**Published:** 2021-06-09

**Authors:** Marco Massani, Luca Bonariol, Tommaso Stecca

**Affiliations:** HPB Hub Reference Center, First General Surgery Unit, Department of Surgery, Azienda ULSS2 Marca Trevigiana, 31100 Treviso, Italy; luca.bonariol@aulss2.veneto.it (L.B.); tommaso.stecca@aulss2.veneto.it (T.S.)

**Keywords:** intrahepatic cholangiocarcinoma, unresectable, hepatic arterial infusion chemotherapy

## Abstract

Cholangiocarcinoma (CCA) is the second most common primitive liver cancer. Despite recent advances in the surgical management, the prognosis remains poor, with a 5-year survival rate of less than 5%. Intrahepatic CCA (iCCA) has a median survival between 18 and 30 months, but if deemed unresectable it decreases to 6 months. Most patients have a liver-confined disease that is considered unresectable because of its localization, with infiltration of vascular structures or multifocality. The peculiar dual blood supply allows the delivery of high doses of chemotherapy via a surgically implanted subcutaneous pump, through the predominant arterial tumor vascularization, achieving much higher and more selective tumor drug levels than systemic administration. The results of the latest studies suggest that adequate and early treatment with the combination approach of hepatic arterial infusion (HAI) and systemic (SYS) chemotherapy is associated with improved progression-free and overall survival than SYS or HAI alone for the treatment of unresectable iCCA. Current recommendations are limited by a lack of prospective trials. Individualization of chemotherapy and regimens based on selective targets in mutant iCCA are a focus for future research. In this paper we present a comprehensive review of the studies published to date and ongoing trials.

## 1. Introduction

The first description of a case of cholangiocarcinoma (CCA) dates back to 1840 by Durand-Fardel [1]. CCA is an epithelial tumor with features of cholangiocyte differentiation [2]. It originates from the ductal epithelium of the biliary tree from the canals of Hering to the main bile duct [3]. Most patients suffer from an unresectable disease from presentation and death occurs within 12 months from diagnosis due to the effects of cachexia, rapid decline in performance status, liver failure and recurrent sepsis [4].

**Figure 1:**
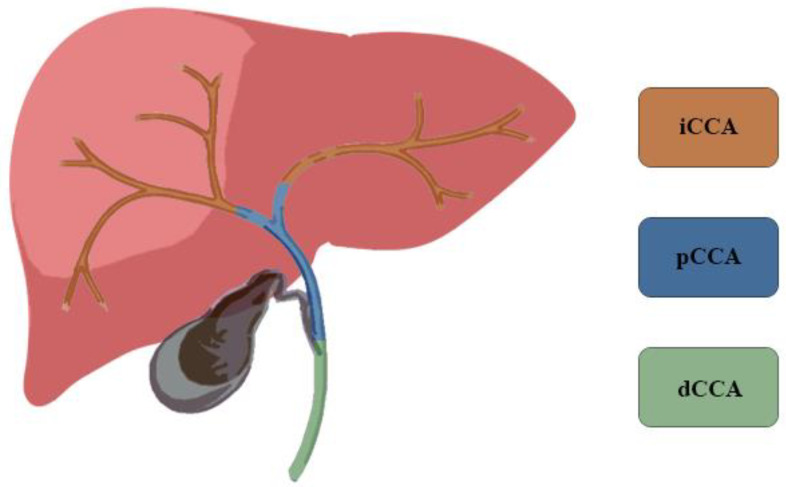
Cholangiocarcinoma is classified according to the anatomical location: intrahepatic (iCCA), perihilar (pCCA) and distal (dCCA).

CCA represents 3% of all gastrointestinal tumors and is the second most common primitive liver cancer, accounting for 15% of all primary liver tumors [5,6]. CCA is classified according to its anatomical location: intrahepatic (iCCA), perihilar (pCCA) and distal CCA (dCCA) (Figure 1). According to the classification of the Liver Cancer Study Group of Japan, iCCA can be further classified by macroscopic growth patterns: mass-forming (MF iCCA), periductal infiltrating (PI iCCA), and intraductal growing (IG iCCA) [3]. In a large series of patients suffering from bile duct cancer, 8% had iCCA, 50% had pCCA, and 42% had dCCA [3,7,8]. The highest incidence is reached in the seventh decade and is slightly more frequent in males with a ratio of 1.5:1 [9]. The incidence rates are characterized by a wide geographical variation, reflecting the distribution of local risk factors in addition to genetic differences among different populations [8,10].

CCA is a rare cancer, but increases in incidence and mortality rates have been documented in the past few decades worldwide [4,11]. In 2007, Welzel et al. reported in the US a 4% annual increase in the incidence of iCCA from 1992 to 2000 [12]. In 2019, Bertuccio et al. extracted data from the World Health Organization and Pan American Health Organization databases for 32 countries from Europe, the Americas, and Australasia demonstrating the global increase in mortality from iCCA from 1995 to 2016 [11]. Recently, Rahib et al. estimated that by 2040, liver and intrahepatic bile duct cancer will surpass colorectal cancer to become the third most common cause of cancer-related death after lung and pancreatic cancer [13].

## 2. Available Treatments

Despite recent advances in the surgical management of this neoplasm, the prognosis remains poor, with a 5-year survival rate of less than 5%. The median survival for iCCA is between 18 and 30 months, but if deemed unresectable it decreases to 6 months. Surgery is the only curative therapeutic option for tumors at the initial stage [14]. However, most patients present with an unresectable, metastatic or locally advanced disease and only 25% are eligible for resection [15,16].

Patients with advanced-stage cholangiocarcinoma are not amenable to locoregional or surgical therapies. The first-line chemotherapy is the combination of cisplatin and gemcitabine (CIS-GEM). In 2010, Valle et al. defined the standard treatment for advanced cholangiocarcinoma in the ABC (Advanced Biliary Cancer)-02 phase III trial. This study provided concrete support for CIS-GEM as compared with gemcitabine alone both in overall survival (11.7 vs. 8.1 months; *p* < 0.001) and in progression-free survival (8.0 vs. 5.0 months; *p* < 0.001) [17]. FOLFOX (folinic acid, fuorouracil and oxaliplatin) can be recommended as the second-line standard of care chemotherapy. The ABC-06 clinical trial demonstrated improvement in OS after progression to CIS-GEM. Although differences in median OS were modest (5.3 versus 6.2 months) between study arms, differences in survival at 6 months (35.5% versus 50.6%) and 12 months (11.4% versus 25.9%) were clinically meaningful [18].

Targeted therapies such as inhibitors of isocitrate dehydrogenase 1 (IDH1) [19,20], fibroblast growth factor receptor (FGFR) [21,22,23,24,25] and tropomyosin receptor kinases (TRK) [26,27] or WNT [28] pathway alterations are currently being tested in patients with iCCA [16]. The final results from ClarIDHy, a phase III study, demonstrated the clinical benefit of ivosidenib (a small-molecule targeted inhibitor of mutated Isocitrate dehydrogenase 1, IDH1) versus placebo in patients with previously treated CCA and IDH1 mutation in terms of progression-free survival (HR 0.37) and median OS (HR 0.69) [20].

A large proportion of patients diagnosed with iCCA in liver-predominant disease could be suitable for liver-directed therapies: trans-arterial radio-embolization with yttrium-90 (TARE), stereotactic body radiotherapy (SBRT) and hepatic arterial infusion chemotherapy (HAI) [29]. TARE is the most developed approach but robust evidence in support is still modest. The SIRCCA clinical trial evaluating TARE followed by CIS-GEM chemotherapy vs. CIS-GEM chemotherapy alone as first-line treatment of patients with unresectable iCCA was prematurely interrupted because of poor recruitment (NCT02807181). Results from prospective studies (ABC-07 study and EudraCT 2014-003656-31) are awaited to evaluate the benefit derived from SBRT in association with systemic chemotherapy.

The last NCCN clinical practice guideline on hepatobiliary cancers (v. 2.2021), gives advice on general patient selection criteria for arterially directed therapies (TARE, SRBT and HAIC) including unresectable or metastatic iCCA, without extra-hepatic disease. HAI chemotherapy is recommended only in the context of a clinical trial or in tertiary referral Hepato-Pancreato-Biliary centers for patients with advanced disease confined to the liver [30].

## 3. Hepatic Arterial Infusion Chemotherapy

### 3.1. Rationale

Most patients suffering from iCCA have a liver-confined disease that is considered unresectable because of its localization, with infiltration of vascular structures or multifocality [31]. The peculiar dual blood supply of the liver allows the delivery of high doses of chemotherapy through the predominant arterial tumor vascularization, reserving the portal blood flow to healthy liver parenchyma. Hepatic extraction and first-pass metabolism (up to 99%) of selected drug regimens diminishes systemic exposure and toxic effects [32], achieving much higher and more selective tumor drug levels than systemic administration [33]. The results of the first small trials conducted in the 1970s and 1980s, together with these assumptions, led to the wide use of hepatic arterial infusion chemotherapy via a surgically implanted subcutaneous pump [34,35,36].

### 3.2. Port Placement Technique

**Figure 2:**
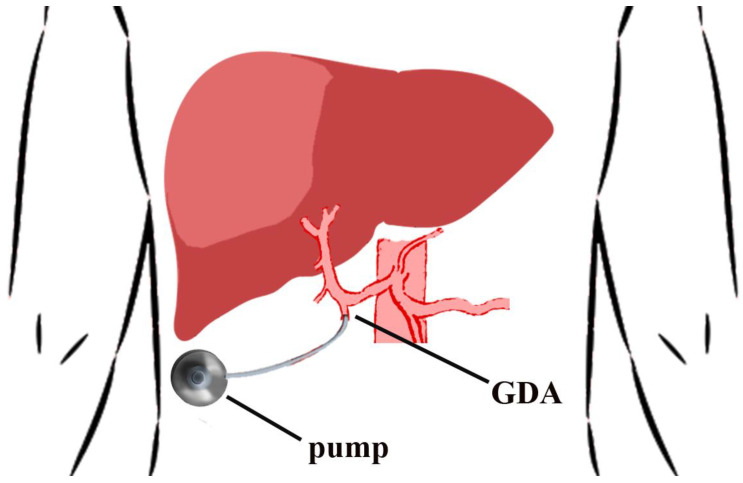
Placement location for hepatic arterial infusion chemotherapy pump. The catheter tip is placed at the origin of the gastroduodenal artery (GDA).

Hepatic vascular anatomy must be evaluated pre-operatively with an arterial phase CT. Up to one third of patients have abnormal hepatic artery anatomy. The catheter tip is placed just at the origin of the gastroduodenal artery (GDA). The tip of the catheter must not create turbulence in the hepatic artery so as to affect the catheter and the cannulated artery long-term patency [37]. Adequate perfusion of the entire hepatic parenchyma must be ensured, without showing any leakage to vicinious organs. Suprapyloric arterial branches and the right gastric artery are ligated to prevent perfusion to the duodenum. Liver-only pump perfusion is assessed by injecting a bolus of methylene blue solution or fluorescein (with a Woods lamp) into the pump [36,38]. All the accessory/replaced vessels are ligated [39,40] relying on hepatic cross perfusion. Hepatic lobar arteries are not end arteries and the occlusion of a variant vessel will not affect the flow from the contralateral hepatic lobe through collateral vessels that prompt develop [37]. Cholecystectomy is performed to avoid chemotherapy-induced cholecystitis. Depending on the infusion pump model that is used, the positioning lodge is made either in the lower left abdomen (stoma is a contraindication), according to the Memorial Sloan Kettering Cancer Center (MSKCC) technique [36], or in the right side of the thoracic wall [38] (Figure 2).

Absolute contraindications to HAI chemotherapy include: poor hepatic function, prolonged systemic chemotherapy, extensive liver tumor burden, portal hypertension, portal vein thrombosis, and hepatic artery occlusion [36].

Assessment of locoregional lymph nodes is routinely performed by hepatic artery, hepatoduodenal, portocaval and peripancreatic lymphadenectomy (stations 8, 12 and 13) as it provides good exposure to the hepatic artery and it determines nodal staging at the time of pump placement, thus resulting in no misclassified patients [36,41]. The role of routine lymphadenectomy for intrahepatic cholangiocarcinoma is still controversial [42]. The AJCC eighth edition [43] and the National Comprehensive Cancer Network [44] recommend a minimum of six harvested lymph nodes for adequate nodal staging. Bagante et al. in 2017 demonstrated that pathological nodal status is strongly associated with long-term outcome and that radiological lymph node staging could be inaccurate in up to 40% of patients [45]. In 2020, Bartsch et al. confirmed that preoperative imaging has a sensitivity of only 71.1% in the detection of suspicious lymph nodes [46]. Lymphadenectomy has been proven to have no benefits to cancer-specific survival for resectable patients, but showed a significant benefit in unresectable patients relieving portal tumor burden and prolonging survival [47], as well as giving the opportunity of a more personalized approach to systemic therapy [48].

### 3.3. Complications

Overall morbidity associated with the pump has been reported as 12% to 41% of patients [49,50,51,52]. Complications include pump malfunction, migration or flipping, pocket infection or hematoma, catheter thrombosis or displacement, catheter erosion, arterial thrombosis or arterial dissection, extrahepatic perfusion, and incomplete perfusion. In 2005, the largest retrospective single center experience to date published by the MSKCC study group evaluated the complications and long-term durability of the pump in 544 patients treated between 1986 and 2001 [52]. Pump-related complications were recorded in 22% of patients, most commonly due to the hepatic arterial system (51%): arterial thrombosis, extrahepatic perfusion, incomplete hepatic perfusion, and hemorrhage. Pump complications were salvaged in 45% of patients and early complications (<30 days) were more likely to be salvaged than late ones (70% vs. 30%) [52]. Even though post-operative pocket infections are unusual (2%), care must be taken to avoid any contamination during the operation and whenever the pump is used for chemotherapy infusion post-operatively. Pump pocket infections are difficult to manage conservatively and re-siting of the pump must be considered [36]. Pump flipping is another problem that can be encountered, especially in obese patients where it is useful to routinely consider the placement in the chest wall. Overall, 12% of patients had a complication that rendered the pump nonfunctional. Long-term durability of pump function is excellent, with a reported incidence of pump failure at 6 months, 1 year, and 2 years after placement of 5%, 9%, and 16%, respectively [52].

Hepatic arterial infusion pump chemotherapy related toxicity includes chemical hepatitis, gastritis, peptic ulcer, and biliary sclerosis (BS). The incidence of BS in patients who received floxuridine-based chemotherapy is between 0.9% and 26% [49,53,54,55,56,57,58,59,60,61,62]. This is a clinically relevant adverse event that may require a biliary stent or result in a chronic liver damage [63]. In these patients it is imperative to routinely monitor biochemical parameters as the first signs of BS are manifested through the elevations in serum alkaline phosphatase and/or total bilirubin. When liver function tests are increased, HAI chemotherapy should be interrupted or the dose reduced. It is common practice to add intra-arterial steroids (dexamethasone 4 mg) to HAI chemotherapy to prevent/reduce BS.

## 4. Literature Review

HAI chemotherapy has been initially developed for colorectal liver metastases treatment [53,64,65], but in the last two decades more data have been published for iCCA treatment (Table 1).

In 2005, a phase two study by Cantore et al. of 30 patients with unresectable iCCA (*n* = 25) or gallbladder adenocarcinoma (*n* = 5) treated with epirubicin and cisplatin HAI as a bolus combined with systemic continuous infusion of 5-fluorouracil (5-FU) demonstrated a 40% overall response rate. The median progression-free survival (PFS) and overall survival (OS) were 7.1 and 13.2 months, respectively, and the 1- and 2-year survival rates were 54% and 20%, respectively. Grade 3 toxicity was observed in 11 of 30 patients treated [66].

In 2009, the MSKCC research group led by Kemeny NE and Jarnagin WR investigated the efficacy of HAI with floxuridine (FUDR) and dexamethasone in 34 patients with unresectable primary liver cancer (26 iCCA and 8 hepatocarcinoma). They demonstrated a response rate of 47.1%, median survival was 29.5 months and the 2-year survival was 67%. Patients with iCCA had a higher response rate (53.8%) compared with those with HCC (25%). Hepatic progression-free survival (HPFS) in iCCA patients was 11.3 months. One patient was converted to resectability, all patients ultimately progressed, and nearly all were treated at some point with systemic therapy. The 1-, 2-, and 3-year survival rates were 88%, 67%, and 29%, respectively. Five patients (14.7%) experienced grade 3 or 4 toxicity [67]. The same research group has published further update studies. In 2011, there was a trial in which 22 patients (18 iCCA and 4 HCC) were treated by systemic (IV) bevacizumab in addition to the previously described HAI regimen. Median OS was 31.1 months (CI 14.14–33.59), PFS was 8.45 months (CI 5.53–11.05), and HPFS was 11.28 months (CI 7.93–15.69). The trial was prematurely terminated due to increased biliary toxicity; 24% patients experienced bilirubin elevation and biliary stents were placed in 13.6% [68]. In 2016, the MSKCC group published a subsequent review of 104 patients with liver-only, unresectable iCCA treated with HAI and systemic (SYS) chemotherapy (*n* = 78) or systemic chemotherapy alone (*n* = 26). They demonstrated a better response rate for patients who received HAI and SYS chemotherapy than the rate for those who received SYS alone. PFS was 12 vs. 7 months for HAI/SYS and SYS, respectively, although not significant *p* = 0.2. OS was 30.8 vs. 18.4 months for HAI/SYS and SYS, respectively. However, the conversion to resectability demonstrated (8/104) is far below the rates achieved for colorectal liver metastases [71]. In 2019, the last phase two clinical trial published included 38 unresectable ICC patients treated with HAI FUDR chemotherapy combined with systemic gemcitabine and oxaliplatin. The median PFS was 11.8 months, the median OS was 25.0 months, and the 1-year OS rate was 89.5%. The results of the study suggest that the combination approach is associated with further improvements in progression-free survival than SYS alone [33].

A phase I/II study published in 2011 by the Japan Interventional Radiology in Oncology Study Group was designed to ascertain the recommended dose (RD) of HAI using gemcitabine (GEM) for iCCA and to assess its efficacy and safety. RD was set at 1000 mg/m^2^. A total of 13 patients were treated with the RD using a percutaneously placed HAI catheter-port system as a 30-min infusion on days 1, 8, and 15 every 4 weeks for 5 cycles. The response rate was 7.7%, below the established threshold efficacy rate of 20%. Complications related to the HAI itself or the implanted catheter-port system occurred in 6 cases (24%) and the incidence of adverse events of Grade 3 or more in all patients treated (*n* = 25) was 48%. The authors concluded that this protocol did not have any advantage over systemic treatment [69].

In 2014 a French retrospective study by Ghiringhelli et al. analyzed the outcome of 12 consecutive patients with unresectable iCCA treated with HAI (percutaneously placed catheter-port system) of GEM (1000 mg/m^2^) followed by systemic oxaliplatin (100 mg/m^2^) as second-line treatment. The overall response rate was 66% and the disease control rate was 91%. The median PFS was 9.2 months (CI 5.1–29.4) and the median OS 9.1 months (CI 13.2–49.7). Six patients (50%) experienced a grade 3/4 toxicity [70].

In 2015, Massani M. et al. published the retrospective experience with HAI treatment alone (fluorouracil and oxaliplatin) in 11 unresectable iCCA patients. A CT scan performed after the sixth cycle of therapy revealed that 5 of them had partial hepatic response, 2 stable disease, and 4 disease progression. The median OS was 17.6 months. Three of the patients with partial hepatic response underwent resection and two had more than 70% tumor necrosis. The median survival of patients with liver-only disease treated with systemic chemotherapy, who were not submitted for resection, was 15.3 months [38].

A pilot study published in 2018 and conducted in Japan between 2007 and 2011 compared 12 patients with unresectable iCCA who received HAI of cisplatin plus oral S-1 to 16 patients who received conventional therapies (systemic or loco-regional). Cisplatin was administered via a catheter placed in the femoral artery and introduced into the hepatic artery under angiographic guidance at each cycle. S-1 is an oral fluoropyrimidine, designed to improve the antitumor activity of 5-fluorouracil (5-FU) concomitantly with an intent to reduce its toxicity [73]. All of the 12 patients completed at least 2 courses of chemotherapy. The median OS was 10.1 months (CI 3.6–23.2). Grade 3 anemia occurred in only 1 patient (4.5%) [72].

With regard to the clinical trials currently in progress, the NCT01525069 trial is a pilot study that enrolled patients with unresectable iCCA that were allocated to three different treatment arms: HAI of FUDR alone or in combination with oxaliplatin and/or gemcitabine. The NCT03771846 promoted by the Cancer Center Sun Yat-sen University aims to evaluate the efficacy and safety of HAI of irinotecan, oxaliplatin, 5-fluorouracil, and leucovorin compared to systemic chemotherapy of gemcitabine and oxaliplatin in patients with unresectable iCCA. The French multicenter phase 2 Trial GEMOXIA-02 (NCT03364530) aims to determine the objective response rate of HAI of gemcitabine/oxaliplatin administered as second-line treatment in patients with non-metastatic unresectable iCCA. The HELIX ICC (NCT04251715) is a phase II trial designed to study the efficacy and safety of systemic induction of mFOLFIRINOX, followed by HAI of FUDR plus dexamethasone administered concurrently with systemic mFOLFIRI in treating patients with liver-dominant unresectable iCCA.

## 5. Conclusions

Early published experiences considered groups of patients with different primary liver cancers and collided with the possible chemotherapy-induced biliary damage. The results of the latest studies suggest that adequate and early treatment with the combination approach of HAI and SYS chemotherapy is associated with improved progression-free and overall survival than SYS or HAI alone for the treatment of unresectable iCCA. However, to date, the conversion to resectability rate demonstrated is far below the rates achieved for colorectal liver metastases. One of the major limitations of HAI chemotherapy is the limited availability of surgeons and oncologists experienced with its use outside of a few referral HPB centers worldwide. Current recommendations for the use of regional therapy in unresectable iCCA are limited by a lack of prospective trials. Rigorous evaluation of these strategies in clinical trials is essential. Individualization of chemotherapy and regimens based on selective targets in mutant iCCA are a focus for future research.

## Figures and Tables

**Table 1:** Summary of studies. HAI +/- SYS chemotherapy treatments for unresectable iCCA.

Study	Number and Type of Patients	Treatment Regimen	PFS	OS
Cantore et al., 2005 [66]	30 BTC •25 iCCA •5 gallbladder cancer	3-week cycle HAI epirubicin 50 mg/m^2^, CIS 60 mg/m^2^ as bolus on Day 1 5-FU 200 mg/m^2^ per day by continuous infusion Day 1 to Day 14	7.1 months (C.I. 1.6–19.1)	13.2 months
Jarnagin et al., 2009 [67]	34 PLC •26 iCCA •8 HCC	4-week cycle HAI: FUDR (0.16 mg/kg × 20/pump flow rate) and DEXA 25 mg on day 1 for 14-days of each cycle	7.4 months	29.5 months
Kemeny et al., 2011 [68]	22 PLC •18 iCCA •4 HCC	4-week cycle HAI: FUDR (0.16 mg/kg × 30/pump flow rate) and DEXA 25 mg on day 1 for 14-days of each cycle SYS: bevacizumab 5 mg/kg every other week.	8.45 months (CI 5.53–11.05)	31.1 months (CI 14.14–33.59)
Inaba et al., 2011 [69]	13 iCCA	4-week cycle HAI: GEM 1000 mg/m^2^ 30-min infusion on days 1, 8, and 15 for 5 cycles	-	389 days (CI 158–620)
Ghiringhelli et al., 2013 [70]	12 iCCA	Second-line treatment 2-week cycle HAI: GEM (1000 mg/m^2^ given over 30 min) followed by OX (100 mg/m^2^ given over 2 h)	9.2 months (CI 2.1–29.4)	20.3 months (CI 13.2–49.7)
Massani et al., 2015 [38]	11 iCCA	2-week cycle HAI: Day 1: 100 mg/mq of OX Day 2: 5 FU 7 mg/kg at 2 mL/h in CI for 48 h	-	17.6 months
Konstantinidis et al., 2016 [71].	104 iCCA •78 HAI/SYS •26 SYS	4-week cycle HAI: FUDR (0.16 mg/kg × 20/pump flow rate) and DEXA 25 mg on day 1 for 14-days of each cycle SYS: mostly GEM based	HAI/SYS 12 months SYS 7 months	HAI/SYS 30.8 months SYS 18.4 months
Higaki et al., 2018 [72]	12 iCCA	42-day cycle HAI: CIS 65 mg/m^2^ 2 mL/min on Day 1 SYS: S-1 60 mg/m^2^ per day 1–28.	-	10.1 months (CI 3.6–23.2)
Cercek A, et al., 2019 [33]	38 iCCA	4-week cycle HAI: FUDR (0.12 mg/kg × 30/pump flow rate) and DEXA 30 mg/pump on day 1 for 14-days of each cycle SYS: GEM (800 mg/m^2^) with OX (85 mg/m^2^) on Day 1 or 15, every 2 weeks	11.8 months (1-sided 90% CI, 11.1)	25.0 months (95% CI, 20.6-not reached)

Table legend: PFS, progression-free survival; OS, overall survival; BTC, biliary tract cancer; PLC, primary liver carcinoma; iCCA, intrahepatic cholangiocarcinoma; HCC, hepatocarcinoma; CI, confidence interval; HAI, hepatic arterial infusion; SYS, systemic; FUDR, floxuridine; GEM, gemcitabine; CIS, cisplatin; OX, oxaliplatin; DEXA, dexamethasone; 5-FU, 5-fluorouracil.

## References

[1] Renshaw K. (1922). Malignant neoplasms of the extrahepatic biliary ducts. Ann. Surg..

[2] Rizvi S., Gores G.J. (2013). Pathogenesis, diagnosis, and management of cholangiocarcinoma. Gastroenterology.

[3] Banales J.M., Cardinale V., Carpino G., Marzioni M., Andersen J.B., Invernizzi P., Lind G.E., Folseraas T., Forbes S.J., Fouassier L. (2016). Expert consensus document: Cholangiocarcinoma: Current knowledge and future perspectives consensus statement from the European Network for the Study of Cholangiocarcinoma (ENS-CCA). Nat. Rev. Gastroenterol. Hepatol..

[4] Khan S.A., Thomas H.C., Davidson B.R., Taylor-Robinson S.D. (2005). Cholangiocarcinoma. Lancet.

[5] Alvaro D., Bragazzi M.C., Benedetti A., Fabris L., Fava G., Invernizzi P., Marzioni M., Nuzzo G., Strazzabosco M., Stroffolini T. (2011). Cholangiocarcinoma in Italy: A national survey on clinical characteristics, diagnostic modalities and treatment. Results from the ‘Cholangiocarcinoma’ committee of the Italian Association for the Study of Liver disease. Dig. Liver Dis..

[6] Khan S.A., Davidson B.R., Goldin R.D., Heaton N., Karani J., Pereira S.P., Rosenberg W.M., Tait P., Taylor-Robinson S.D., Thillainayagam A.V. (2012). Guidelines for the diagnosis and treatment of cholangiocarcinoma: An update. Gut.

[7] DeOliveira M.L., Cunningham S.C., Cameron J.L., Kamangar F., Winter J.M., Lillemoe K.D., Choti M.A., Yeo C.J., Schulick R.D. (2007). Cholangiocarcinoma: Thirty-one-year experience with 564 patients at a single institution. Ann. Surg..

[8] Nakeeb A., Pitt H.A., Sohn T.A., Coleman J.A., Abrams R.A., Piantadosi S., Hruban R.H., Lillemoe K.D., Yeo C.J., Cameron J.L. (1996). Cholangiocarcinoma: A spectrum of intrahepatic, perihilar, and distal tumors. Ann Surg..

[9] Aljiffry M., Walsh M.J., Molinari M. (2009). Advances in diagnosis, treatment and palliation of cholangiocarcinoma: 1990–2009. World J. Gastroenterol..

[10] Ebata T., Kosuge T., Hirano S., Unno M., Yamamoto M., Miyazaki M., Kokudo N., Miyagawa S., Takada T., Nagino M. (2014). Proposal to modify the International Union Against Cancer staging system for perihilar cholangiocarcinomas. Br. J. Surg..

[11] Bertuccio P., Malvezzi M., Carioli G., Hashim D., Boffetta P., El-Serag H.B., La Vecchia C., Negri E. (2019). Global trends in mortality from intrahepatic and extrahepatic cholangiocarcinoma. J. Hepatol..

[12] Welzel T.M., Mellemkjaer L., Gloria G., Sakoda L.C., Hsing A.W., El Ghormli L., Olsen J.H., McGlynn K.A. (2007). Risk factors for intrahepatic cholangiocarcinoma in a low-risk population: A nationwide case-control study. Int. J. Cancer.

[13] Rahib L., Wehner M.R., Matrisian L.M., Nead K.T. (2021). Estimated Projection of US Cancer Incidence and Death to 2040. JAMA Netw. Open.

[14] Sempoux C., Jibara G., Ward S.C., Fan C., Qin L., Roayaie S., Fiel M.I., Schwartz M., Thung S.N. (2011). Intrahepatic cholangiocarcinoma: New insights in Pathology. Semin. Liver Dis..

[15] Mosconi S., Beretta G.D., Labianca R., Zampino M.G., Gatta G., Heinemann V. (2009). Cholangiocarcinoma. Crit. Rev. Oncol. Hematol..

[16] Banales J.M., Marin J.J.G., Lamarca A., Rodrigues P.M., Khan S.A., Roberts L.R., Cardinale V., Carpino G., Andersen J.B., Braconi C. (2020). Cholangiocarcinoma 2020: The next horizon in mechanisms and management. Nat. Rev. Gastroenterol. Hepatol..

[17] Valle J., Wasan H., Palmer D.H., Cunningham D., Anthoney A., Maraveyas A., Madhusudan S., Iveson T., Hughes S., Pereira S.P. (2010). Cisplatin plus Gemcitabine versus Gemcitabine for Biliary Tract Cancer. N. Engl. J. Med..

[18] Lamarca A., Palmer D.H., Wasan H.S., Ross P.J., Ma Y.T., Arora A., Falk S., Gillmore R., Wadsley J., Patel K. (2019). ABC-06|A randomised phase III, multi-centre, open-label study of active symptom control (ASC) alone or ASC with oxaliplatin / 5-FU chemotherapy (ASC+mFOLFOX) for patients (pts) with locally advanced/metastatic biliary tract cancers (ABC) previously-tr. J. Clin. Oncol..

[19] Lowery M.A., Abou-Alfa G.K., Burris H.A., Janku F., Shroff R.T., Cleary J.M., Azad N.S., Goyal L., Maher E.A., Gore L. (2017). Phase I study of AG-120, an IDH1 mutant enzyme inhibitor: Results from the cholangiocarcinoma dose escalation and expansion cohorts. J. Clin. Oncol..

[20] Zhu A.X., Macarulla T., Javle M.M., Kelley R.K., Lubner S.J., Adeva J., Cleary J.M., Catenacci D.V.T., Borad M.J., Bridgewater J.A. (2021). Final results from ClarIDHy, a global, phase III, randomized, double-blind study of ivosidenib (IVO) versus placebo (PBO) in patients (pts) with previously treated cholangiocarcinoma (CCA) and an isocitrate dehydrogenase 1 (IDH1) mutation. J. Clin. Oncol..

[21] Javle M., Lowery M., Shroff R.T., Weiss K.H., Springfeld C., Borad M.J., Ramanathan R.K., Goyal L., Sadeghi S., Macarulla T. (2018). Phase II Study of BGJ398 in Patients With FGFR-Altered Advanced Cholangiocarcinoma. J. Clin. Oncol. Off. J. Am. Soc. Clin. Oncol..

[22] Javle M.M., Shroff R.T., Zhu A., Sadeghi S., Choo S., Borad M.J., Lowery M.A., El-Khoueiry A., Macarulla T., Philip P.A. (2016). A phase 2 study of BGJ398 in patients (pts) with advanced or metastatic FGFR-altered cholangiocarcinoma (CCA) who failed or are intolerant to platinum-based chemotherapy. J. Clin. Oncol..

[23] Mazzaferro V., Shaib W., Rimassa L., Harris W., Personeni N., El-Rayes B., Tolcher A., Hall T., Wang Y., Schwartz B. (2016). PD-019 ARQ 087, an oral pan- fibroblast growth factor receptor (FGFR) inhibitor, in patients (pts) with advanced and/or metastatic intrahepatic cholangiocarcinoma (iCCA). Ann. Oncol..

[24] Tran B., Meric-Bernstam F., Arkenau H.-T., Bahleda R., Kelley R.K., Hierro C., Ahn D., Zhu A., Javle M., Winkler R. (2018). Efficacy of TAS-120, an irreversible fibroblast growth factor receptor inhibitor (FGFRi), in patients with cholangiocarcinoma and FGFR pathway alterations previously treated with chemotherapy and other FGFRi’s. Ann. Oncol..

[25] Vogel A., Sahai V., Hollebecque A., Vaccaro G., Melisi D., Al-Rajabi R., Paulson A.S., Borad M.J., Gallinson D., Murphy A.G. (2019). FIGHT-202: A phase II study of pemigatinib in patients (pts) with previously treated locally advanced or metastatic cholangiocarcinoma (CCA). Ann. Oncol..

[26] Drilon A., Siena S., Ou S.-H.I., Patel M., Ahn M.J., Lee J., Bauer T.M., Farago A.F., Wheler J.J., Liu S.V. (2017). Safety and Antitumor Activity of the Multitargeted Pan-TRK, ROS1, and ALK Inhibitor Entrectinib: Combined Results from Two Phase I Trials (ALKA-372-001 and STARTRK-1). Cancer Discov..

[27] Drilon A., Laetsch T.W., Kummar S., DuBois S.G., Lassen U.N., Demetri G.D., Nathenson M., Doebele R.C., Farago A.F., Pappo A.S. (2018). Efficacy of Larotrectinib in TRK Fusion–Positive Cancers in Adults and Children. N. Engl. J. Med..

[28] Ong C.K., Subimerb C., Pairojkul C., Wongkham S., Cutcutache I., Yu W., McPherson J.R., Allen G.E., Ng C.C., Wong B.H. (2012). Exome sequencing of liver fluke-associated cholangiocarcinoma. Nat. Genet..

[29] Koay E.J., Odisio B.C., Javle M., Vauthey J.-N., Crane C.H. (2017). Management of unresectable intrahepatic cholangiocarcinoma: How do we decide among the various liver-directed treatments?. Hepatobiliary Surg. Nutr..

[30] Hepatobiliary Cancer (2021). National Comprehensive Cancer Network. https://www.nccn.org/professionals/physician_gls/pdf/hepatobiliary.pdf.

[31] Nathan H., Aloia T.A., Vauthey J.N., Abdalla E.K., Zhu A.X., Schulick R.D., Choti M.A., Pawlik T.M. (2009). A proposed staging system for intrahepatic cholangiocarcinoma. Ann. Surg. Oncol..

[32] Ensminger W.D., Gyves J.W. (1983). Clinical pharmacology of hepatic arterial chemotherapy. Semin. Oncol..

[33] Cercek A., Boerner T., Tan B.R., Chou J.F., Gönen M., Boucher T.M., Hauser H.F., Do R.K.G., Lowery M.A., Harding J.J. (2020). Assessment of Hepatic Arterial Infusion of Floxuridine in Combination with Systemic Gemcitabine and Oxaliplatin in Patients with Unresectable Intrahepatic Cholangiocarcinoma: A Phase 2 Clinical Trial. JAMA Oncol..

[34] Johnson L.P., Rivkin S.E. (1985). The implanted pump in metastatic colorectal cancer of the liver. Risk versus benefit. Am. J. Surg..

[35] Weiss G.R., Garnick M.B., Osteen R.T., Steele G.D.J., Wilson R.E., Schade D., Kaplan W.D., Boxt L.M., Kandarpa K., Mayer R.J. (1983). Long-term hepatic arterial infusion of 5-fluorodeoxyuridine for liver metastases using an implantable infusion pump. J. Clin. Oncol. Off. J. Am. Soc. Clin. Oncol..

[36] Thiels C.A., D’Angelica M.I. (2020). Hepatic artery infusion pumps. J. Surg. Oncol..

[37] Allen P.J., Stojadinovic A., Ben-Porat L., Gonen M., Kooby D., Blumgart L., Paty P., Fong Y. (2002). The management of variant arterial anatomy during hepatic arterial infusion pump placement. Ann. Surg. Oncol..

[38] Massani M., Nistri C., Ruffolo C., Bonariol R., Pauletti B., Bonariol L., Caratozzolo E., Morana G., Bassi N. (2015). Intrahepatic chemotherapy for unresectable cholangiocarcinoma: Review of literature and personal experience. Updates Surg..

[39] Cohen A.M., Higgins J., Waltman A.C., Athanasoulis C., McKusick K. (1987). Effect of ligation of variant hepatic arterial structures on the completeness of regional chemotherapy infusion. Am. J. Surg..

[40] Rayner A.A., Kerlan R.K., Stagg R.J., Price D.C., Hohn D.C. (1986). Total hepatic arterial perfusion after occlusion of variant lobar vessels: Implications for hepatic arterial chemotherapy. Surgery.

[41] Jolissaint J.S., Soares K.C., Seier K.P., Kundra R., Gonen M., Shin P.J., Boerner T., Sigel C., Madupuri R., Vakiani E. (2021). Intrahepatic Cholangiocarcinoma with Lymph Node Metastasis: Treatment-Related Outcomes and the Role of Tumor Genomics in Patient Selection. Clin. Cancer Res. Off. J. Am. Assoc. Cancer Res..

[42] Zhou R., Lu D., Li W., Tan W., Zhu S., Chen X., Min J., Shang C., Chen Y. (2019). Is lymph node dissection necessary for resectable intrahepatic cholangiocarcinoma? A systematic review and meta-analysis. Int. Hepato-Pancreato-Biliary Assoc..

[43] Amin M.B., Edge S., Greene F., Byrd D.R., Brookland R.K., Washington M.K., Gershenwald J.E., Compton C.C., Hess K.R., Sullivan D.C. (2017). AJCC Cancer Staging Manual.

[44] Zhang X.-F., Chen Q., Kimbrough C.W., Beal E.W., Lv Y., Chakedis J., Dillhoff M., Schmidt C., Cloyd J., Pawlik T.M. (2018). Lymphadenectomy for Intrahepatic Cholangiocarcinoma: Has Nodal Evaluation Been Increasingly Adopted by Surgeons over Time?A National Database Analysis. J. Gastrointest. Surg. Off. J. Soc. Surg. Aliment. Tract..

[45] Spolverato G., Bagante F., Weiss M., Alexandrescu S., Marques H.P., Aldrighetti L., Maithel S.K., Pulitano C., Bauer T.W., Shen F. (2017). Comparative performances of the 7th and the 8th editions of the American Joint Committee on Cancer staging systems for intrahepatic cholangiocarcinoma. J. Surg. Oncol..

[46] Bartsch F., Hahn F., Müller L., Baumgart J., Hoppe-Lotichius M., Kloeckner R., Lang H. (2020). Relevance of suspicious lymph nodes in preoperative imaging for resectability, recurrence and survival of intrahepatic cholangiocarcinoma. BMC Surg..

[47] Liu J., Zhong M., Feng Y. (2019). Prognostic Factors and Treatment Strategies for Intrahepatic Cholangiocarcinoma from 2004 to 2013: Population-Based SEER Analysis. Transl. Oncol..

[48] Cloyd J.M., Ejaz A., Pawlik T.M. (2020). The Landmark Series: Intrahepatic Cholangiocarcinoma. Ann. Surg. Oncol..

[49] Daly J.M., Kemeny N., Oderman P., Botet J. (1984). Long-term hepatic arterial infusion chemotherapy. Anatomic considerations, operative technique, and treatment morbidity. Arch. Surg..

[50] Curley S.A., Chase J.L., Roh M.S., Hohn D.C. (1993). Technical considerations and complications associated with the placement of 180 implantable hepatic arterial infusion devices. Surgery.

[51] Heinrich S., Petrowsky H., Schwinnen I., Staib-Sebler E., Gog C., El-Ganainy A., Gutt C., Müller H.H., Lorenz M. (2003). Technical complications of continuous intra-arterial chemotherapy with 5-fluorodeoxyuridine and 5-fluorouracil for colorectal liver metastases. Surgery.

[52] Allen P.J., Nissan A., Picon A.I., Kemeny N., Dudrick P., Ben-Porat L., Espat J., Stojadinovic A., Cohen A.M., Fong Y. (2005). Technical complications and durability of hepatic artery infusion pumps for unresectable colorectal liver metastases: An institutional experience of 544 consecutive cases. J. Am. Coll. Surg..

[53] Kemeny N.E., Niedzwiecki D., Hollis D.R., Lenz H.-J., Warren R.S., Naughton M.J., Weeks J.C., Sigurdson E.R., Herndon J.E., Zhang C. (2006). Hepatic arterial infusion versus systemic therapy for hepatic metastases from colorectal cancer: A randomized trial of efficacy, quality of life, and molecular markers (CALGB 9481). J. Clin. Oncol. Off. J. Am. Soc. Clin. Oncol..

[54] Kemeny M.M., Battifora H., Blayney D.W., Cecchi G., Goldberg D.A., Leong L.A., Margolin K.A., Terz J.J. (1985). Sclerosing cholangitis after continuous hepatic artery infusion of FUDR. Ann. Surg..

[55] Kemeny N., Daly J., Reichman B., Geller N., Botet J., Oderman P. (1987). Intrahepatic or systemic infusion of fluorodeoxyuridine in patients with liver metastases from colorectal carcinoma. A randomized trial. Ann. Intern. Med..

[56] Chang A.E., Schneider P.D., Sugarbaker P.H., Simpson C., Culnane M., Steinberg S.M. (1987). A prospective randomized trial of regional versus systemic continuous 5-fluorodeoxyuridine chemotherapy in the treatment of colorectal liver metastases. Ann. Surg..

[57] Hohn D.C., Stagg R.J., Friedman M.A., Hannigan J.F.J., Rayner A., Ignoffo R.J., Acord P., Lewis B.J. (1989). A randomized trial of continuous intravenous versus hepatic intraarterial floxuridine in patients with colorectal cancer metastatic to the liver: The Northern California Oncology Group trial. J. Clin. Oncol. Off. J. Am. Soc. Clin. Oncol..

[58] Martin J.K.J., O’Connell M.J., Wieand H.S., Fitzgibbons R.J.J., Mailliard J.A., Rubin J., Nagorney D.M., Tschetter L.K., Krook J.E. (1990). Intra-arterial floxuridine vs systemic fluorouracil for hepatic metastases from colorectal cancer. A randomized trial. Arch. Surg..

[59] Rougier P., Laplanche A., Huguier M., Hay J.M., Ollivier J.M., Escat J., Salmon R., Julien M., Roullet Audy J.C., Gallot D. (1992). Hepatic arterial infusion of floxuridine in patients with liver metastases from colorectal carcinoma: Long-term results of a prospective randomized trial. J. Clin. Oncol. Off. J. Am. Soc. Clin. Oncol..

[60] Lorenz M., Müller H.H. (2000). Randomized, multicenter trial of fluorouracil plus leucovorin administered either via hepatic arterial or intravenous infusion versus fluorodeoxyuridine administered via hepatic arterial infusion in patients with nonresectable liver metastases from color. J. Clin. Oncol. Off. J. Am. Soc. Clin. Oncol..

[61] Kemeny M.M., Adak S., Gray B., Macdonald J.S., Smith T., Lipsitz S., Sigurdson E.R., O’Dwyer P.J., Benson A.B. (2002). Combined-modality treatment for resectable metastatic colorectal carcinoma to the liver: Surgical resection of hepatic metastases in combination with continuous infusion of chemotherapy—An intergroup study. J. Clin. Oncol. Off. J. Am. Soc. Clin. Oncol..

[62] Martin R.C.G., Edwards M.J., McMasters K.M. (2004). Morbidity of adjuvant hepatic arterial infusion pump chemotherapy in the management of colorectal cancer metastatic to the liver. Am. J. Surg..

[63] Ito K., Ito H., Kemeny N.E., Gonen M., Allen P.J., Paty P.B., Fong Y., Dematteo R.P., Blumgart L.H., Jarnagin W.R. (2012). Biliary sclerosis after hepatic arterial infusion pump chemotherapy for patients with colorectal cancer liver metastasis: Incidence, clinical features, and risk factors. Ann. Surg. Oncol..

[64] Datta J., Narayan R.R., Kemeny N.E., D’Angelica M.I. (2019). Role of Hepatic Artery Infusion Chemotherapy in Treatment of Initially Unresectable Colorectal Liver Metastases: A Review. JAMA Surg..

[65] Kerr D.J., McArdle C.S., Ledermann J., Taylor I., Sherlock D.J., Schlag P.M., Buckels J., Mayer D., Cain D., Stephens R.J. (2003). Intrahepatic arterial versus intravenous fluorouracil and folinic acid for colorectal cancer liver metastases: A multicentre randomised trial. Lancet.

[66] Cantore M., Mambrini A., Fiorentini G., Rabbi C., Zamagni D., Caudana R., Pennucci C., Sanguinetti F., Lombardi M., Nicoli N. (2005). Phase II study of hepatic intraarterial epirubicin and cisplatin, with systemic 5-fluorouracil in patients with unresectable biliary tract tumors. Cancer.

[67] Jarnagin W.R., Schwartz L.H., Gultekin D.H., Gönen M., Haviland D., Shia J., D’Angelica M., Fong Y., DeMatteo R., Tse A. (2009). Regional chemotherapy for unresectable primary liver cancer: Results of a phase II clinical trial and assessment of DCE-MRI as a biomarker of survival. Ann. Oncol..

[68] Kemeny N.E., Schwartz L., Gönen M., Yopp A., Gultekin D., D’Angelica M., Fong Y., Haviland D., Gewirtz A.N., Allen P. (2011). Treating primary liver cancer with hepatic arterial infusion of floxuridine and dexamethasone: Does the addition of systemic bevacizumab improve results?. Oncology.

[69] Inaba Y., Arai Y., Yamaura H., Sato Y., Najima M., Aramaki T., Sone M., Kumada T., Tanigawa N., Anai H. (2011). Phase I/II study of hepatic arterial infusion chemotherapy with gemcitabine in patients with unresectable intrahepatic cholangiocarcinoma (JIVROSG-0301). Am. J. Clin. Oncol. Cancer Clin. Trials..

[70] Ghiringhelli F., Lorgis V., Vincent J., Ladoire S., Guiu B. (2014). Hepatic arterial infusion of gemcitabine plus oxaliplatin as second-line treatment for locally advanced intrahepatic cholangiocarcinoma: Preliminary experience. Chemotherapy.

[71] Konstantinidis I.T., Koerkamp B.G., Do R.K.G., Gönen M., Fong Y., Allen P.J., D’Angelica M., Kingham T.P., DeMatteo R.P., Klimstra D.S. (2016). Unresectable intrahepatic cholangiocarcinoma: Systemic plus hepatic arterial infusion chemotherapy is associated with longer survival in comparison with systemic chemotherapy alone. Cancer.

[72] Higaki T., Aramaki O., Moriguchi M., Nakayama H., Midorikawa Y., Takayama T. (2018). Arterial infusion of cisplatin plus S-1 against unresectable intrahepatic cholangiocarcinoma. Biosci. Trends..

[73] Saif M.W., Syrigos K.N., Katirtzoglou N.A. (2009). S-1: A promising new oral fluoropyrimidine derivative. Expert. Opin. Investig. Drugs.

